# Prebiotics mannan-oligosaccharides accelerate sexual maturity in rats: A randomized preclinical study

**DOI:** 10.14202/vetworld.2021.1210-1219

**Published:** 2021-05-20

**Authors:** Luiz Eduardo Rodrigues, Milena Miyoshi Kishibe, Rogeria Keller, Heliard Rodrigues dos Santos Caetano, Marcos Natal Rufino, Osimar de Carvalho Sanches, Ines Cristina Giometti, Rogério Giuffrida, Hermann Bremer-Neto

**Affiliations:** 1 Department of Functional Sciences, Laboratory of Physiology and Biophysics, Faculty of Medicine, Universidade do Oeste Paulista, Presidente Prudente, São Paulo, Brazil; 2 Department of Functional Sciences, Laboratory of Microbiology, Faculty of Biological Sciences, Universidade do Oeste Paulista, Presidente Prudente, São Paulo, Brazil; 3 Department of Functional Sciences, Laboratory of Physiology, Faculty of Physiotherapy, Universidade do Oeste Paulista, Presidente Prudente, São Paulo, Brazil; 4 Department of Functional Sciences, Laboratory of Physiology, Faculty of Medicine, Universidade do Oeste Paulista, Presidente Prudente, São Paulo, Brazil; 5 Department of Pathology, Faculty of Veterinary Medicine, Universidade de Santo Amaro, São Paulo, Brazil; 6 Department of Reproduction, Faculty of Veterinary Medicine, Universidade do Oeste Paulista, Presidente Prudente, São Paulo, Brazil; 7 Department of Statistics, Faculty of Veterinary Medicine, Universidade do Oeste Paulista, São Paulo, Brazil

**Keywords:** corticosterone, murine, prebiotic, reproductive system, testosterone, yeast

## Abstract

**Background and Aim:**

The prebiotics, mannan-oligosaccharides (MOS), demonstrate the ability to increase probiotic microorganisms and fixation and removal of pathogens associated with chronic systemic inflammation in the digestive system. Inflammatory processes play an important role in modulating the brain-intestinal axis, including maintaining male reproductive function and spermatogenesis and regulating stress. The aim of the present study was to evaluate the action of MOS on testosterone and corticosterone concentrations and the reproductive system development of rats in the growth phase as an animal model.

**Materials and Methods:**

In total, 128 male rats were used, randomly divided into four experimental groups (n=32): Control; MOS 1; MOS 2; and MOS 3. From each group, eight animals were sacrificed in four experimental moments (14, 28, 42, and 56 days, respectively, moments 1, 2, 3, and 4) and hormonal measurements and histological evaluations were performed.

**Results:**

The results revealed the effect of diet, MOS, and timing on testicle weight (p<0.05). At moments 3 and 4, the groups supplemented with MOS showed higher concentrations of testosterone and decreased corticosterone levels throughout the experimental period. Groups supplemented with MOS showed an increase in the frequency of relative sperm and sperm scores. The radii of the seminiferous tubules presented a significant statistical effect of the diet, moments, and diet + moment interaction.

**Conclusion:**

It was concluded that the three different MOS prebiotics brought forward sexual maturity.

## Introduction

The prebiotics, mannan-oligosaccharides (MOS), are mostly complex molecules that are linked to portions of proteins derived from the cell wall of the yeast *Saccharomyces cerevisiae* [1] and their production originates mainly from the sugar and alcohol, beer, and bakery sectors [2]. These functional foods, undigested by enzymes, salts, and acids produced by the body, are selectively fermented by microorganisms of the gastrointestinal (GI) tract [3]. Supplementation with prebiotics has been studied due to their ability to increase probiotic microorganisms, especially the genera *Bifidobacterium* and *Lactobacillus*, beneficially modulating the native microbiota [4], as well as increasing short-chain fatty acid (SCFAs) production, decreasing intestinal pH, and beneficially modulating immune, neurobehavioral, and metabolic functions in animals and humans [5].

When supplemented in the appropriate dose and frequency, prebiotics confer immediate beneficial effects associated with the fixation and removal of possible pathogens in the digestive system [6]. These pathogenic agents containing endotoxins are found at very high levels in the mammalian intestine, inducing chronic systemic inflammation [7]. Inflammatory processes play an important role in the etiology of various diseases, and involve multiple pathophysiological systems in the modulation of the brain-intestinal axis, among which is the endocrine system, notably, and gonadal hormones [8,9]. The androgens, testosterone, and corticosterone are steroid hormones involved in several processes, including maintaining male reproductive function, spermatogenesis, and stress regulation [10,11], while estrogens play an important endocrine role in the physiology and pathology of the GI tract, including the regulation of motor and sensory function [12]. Low testosterone levels are associated with an increase in pro-inflammatory factors and the administration of testosterone reduces the levels of these factors, reinforcing the anti-inflammatory effect of testosterone. This indicates that the uncontrolled inflammatory responses in the host contribute to the testicular atrophy phenotype in older mice [13].

A study using prebiotic and probiotic supplementation in the diet reported improvements in the volume of the ejaculate, the concentration of spermatozoa, the number of ejaculated spermatozoa, motility, and the percentage of typical forms, when administered to patients with idiopathic oligoasthenoteratospermia. In addition, supplementation increased follicle-stimulating hormone (FSH), LH, and blood testosterone levels [13].

Therefore, the objective of the present study was to evaluate the action of different MOS on the plasma concentrations of testosterone and corticosterone, as well as the development of the reproductive system, using histomorphometric parameters of reproductive organs of rats in the growth phase, as an animal model.

## Materials and Methods

### Ethical approval

This study was carried out after approval from the Ethics Committee on Animal Use (Protocol no. 1175 and 1177), from the Universidade do Oeste Paulista, Presidente Prudente, SP, Brazil.

### Study period and location

The study was carried out from July 2017 to June 2018 at the Biotério Experimental and laboratory analyses were carried out in the Department of Functional Sciences at the Universidade do Oeste Paulista, located in Presidente Prudente, State of São Paulo (Brazil).

### Animals, care and experimental design

**Figure-1:**
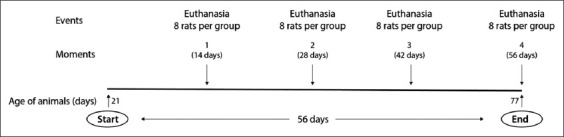
Experiment timeline.

One hundred and twenty-eight male Wistar rats, 21 days of age, with a mean initial body mass of 46.17±4.99 g, were kept in individual cages, under the same standard lighting conditions (light/dark cycle of 12/12 h) and controlled temperature of 22±1°C. The diets and drinking water were provided at will during the 56 days of the experimental period (Figure-1). Concealment of allocation, treatment, and animal management strategy, blinding during and after the intervention, and concealment of evaluations and analysis of results were performed to reduce bias in the study [14].

Four die were formulated (Table-1), comprising the treatments: Control (C), basal diet (SD); MOS1, SD diet supplemented with 1% of the MOS 1 prebiotic (MOS derived from the cell wall of the yeast *S. cerevisiae*, strain 1026, composed of 17% a-mannan [Bio-Mos, Alltech Inc., Brazil]); MOS 2, SD diet supplemented with 0.4% of the prebiotic MOS 2 (Active fraction, a-1,3 and a-1,6 mannanoprotein, derived from a mannan oligosaccharide, presenting 30% a-mannan [*S. cerevisiae*, Alltech Inc., Brazil]); and MOS 3, SD diet supplemented with 1% of the MOS 3 prebiotic (b-glucans, mannans, mannose polymers, chitins [glycosamine polymers], and galactans [galactose polymers] derived from the yeast strain *S. cerevisiae* [ImmunoWall®, ICC Brazil]). The dosages of prebiotics supplemented in the diets were as recommended by the manufacturers. The animals were randomly assigned to the four experimental groups (n=32) using a sequence table generated by the R program [15].

**Table-1:** Composition of ingredients and nutrients of control and experimental diets.^†^

Ingredient (g/100 g)	Composition of the diet (%)^‡^			

	Control	MOS1	MOS2	MOS3
Ground corn	22.18	22.18	22.18	22.18
Ground wheat	22.26	22.26	22.26	22.26
Wheat bran	15.00	15.00	15.00	15.00
Soy bran (49% protein)	5.00	5.00	5.00	5.00
Fish flour (60% protein)	4.00	4.00	4.00	4.00
Alfalfa flour (17% protein)	7.50	7.50	7.50	7.50
Oat hulls	8.50	8.50	8.50	8.50
Purified cellulose	5.50	4.50	5.46	5.40
Corn oil	3.00	3.00	3.00	3.00
Soy oil	3.00	3.00	3.00	3.00
Dry brewer’s yeast	1.00	1.00	1.00	1.00
Sodium chloride	0.30	0.30	0.30	0.30
Calcium phosphate	0.40	0.40	0.40	0.40
Calcium carbonate	0.90	0.90	0.90	0.90
Choline chloride (70% choline)	0.26	0.26	0.26	0.26
Methionine	0.20	0.20	0.20	0.20
MOS 1	-	1.00	-	-
MOS 2	-	-	0.40	-
MOS 3	-	-	-	1.00
Vitamin premix^§^	0.50	0.50	0.50	0.50
Mineral premix^¶^	0.50	0.50	0.50	0.50
Total	100	100	100	100
Analyzed composition				
Protein (%)	15.01	14.96	14.97	14.98
Fat (%)	8.32	14.96	14.97	14.98
Fiber (%)	9.28	9.32	9.22	9.25
Carbohydrates (%)	52	52	52	52
Energy (Kcal/g)	3.39	3.40	3.40	3.39

† Adapted from Rao (1996);

‡ Control and experimental diets/groups: Control (C): Basal diet (SD); MOS 1, SD diet supplemented with 1% of the MOS 1; MOS 2, SD diet supplemented with 4 g.Kg^-1^ of the MOS 2; and MOS 3, SD diet supplemented with 10 g.Kg^-1^ of the MOS 3.

§ Vitamin mix/Kg: Nicotinic acid, 30 mg; pantothenate, 15 mg; pyridoxine, 6 mg; thiamine, 5 mg; riboflavin, 6 mg; folic acid, 2 mg; biotin, 0.2 mg; Vitamin B_12_, 25 mg; Vitamin E, 75 IU; Vitamin A, 4000 IU; Vitamin D_3_, 1000 IU; Vitamin K, 900 mg; Choline, 1000 mg.; and

¶ Mineral mix mg/Kg: Calcium, 5000; Phosphorus, 1.561; Potassium, 3600; Sulfur, 300; Sodium, 1019; Chlorine, 1.574; Magnesium, 507; Ferro, 35; Zinc, 30; Manganese, 10; Copper, 6; Iodine, 0.2; Molybdenum, 0.15; Selenium, 0.15.

MOS: Mannan-oligosaccharides

### Euthanasia and collection of samples

Water and diet consumptions were measured every 3 days and body weight was measured weekly. At each moment: 1, 2, 3, and 4 (respectively, 14, 28, 42, and 56 days after the beginning of the experimental period), eight animals from each of the four experimental groups were anesthetized intraperitoneally, with a dosage of 30 mg.Kg^-1^ of live weight of Tiopental 50 mg.mL^-1^ (Thiopentax, Cristália – Produtos Químicos Farmacêuticos Ltda. – São Paulo/SP, Brazil) [16]. Blood samples were collected through cardiac puncture, centrifuged to obtain plasma, and later transferred to microtubes and stored in a freezer at -80°C. After blood collection, the animals were euthanized by exsanguination [17]. After death, the organs (testicles and adrenal glands) were removed and weighed on a digital analytical scale (Shimadzu - model AY220, Japan).

### Determination of plasma levels of corticosterone and testosterone and histological analysis of the testicles and adrenals

Circulating plasma corticosterone levels were measured in duplicate using the DPC-MedLab commercial solid-phase Coat-A-Count® Rat-Corticosterone Kit (Ref. TKRC1-100 tubes; Los Angeles-California - Appendix A) according to the manufacturer’s instructions. Total plasma testosterone was assayed using a direct plasma radioimmunoassay technique (Coat-a-Count, DPC, Los Angeles, USA). The lower limit of sensitivity was 0.15 nmol/L, and the assay coefficient of variation was 10.4%. The reading was performed by the Gama Counter® counting device - Cobra II (Packard BioScience Company), at the Hormonal Dosing Laboratory - Department of Endocrinology - Faculdade de Medicina Veterinária - UNESP - Araçatuba - Brazil.

The organs (testicles and adrenal glands) were fixed in a modified Davidson solution [18] for approximately 24 h and then washed in running water and transferred to a 70% alcohol solution. The testicles and adrenal glands were embedded in paraffin and 5 mm thick semi-serial sections were obtained using a microtome (Rotatory Microtome, Leica, RM 2265, Germany). Histological sections were stained using the hematoxylin and eosin method. Thirty photographs were taken per organ using a Leica microscope (model DM750, Germany) coupled to a video camera, which sends digital images to a computer equipped with the image analysis program, Image Pro-plus (Media Cybernetics, Silver Spring, Maryland, USA). The relative scores were classified from 0 to 3, from 30 microscopic fields, with 0=absence in 30 fields; 1=mild (1 to 10 out of 30 fields); 2=moderate (11-20 out of 30 fields); and 3=accentuated (>20 out of 30 fields) and were transformed into frequency (%).

### Statistical analysis

The data of cumulative food intake (CFI), mean water consumption (WC), percentage weight gain (WG), and plasma testosterone concentrations were subjected to analysis of variance, followed by the Tukey test. For plasma corticosterone concentrations, simple linear regressions were adjusted, where time in days was considered as an independent variable and plasma dosages as dependent variables. Analysis of variance was performed to assess whether the resulting lines met the conditions of linearity, that is, the slope is different from zero and there is no lack of adjustment. The differences among the slopes of the straight lines of the four treatments/diets were analyzed by the Student’s t-test. The combinations of diet and moments for testicular and adrenal glands weights and radii of the seminiferous tubules were analyzed by two-way analysis of variance with a Tukey *post hoc* test for main effects (diet and moments) and interaction (diet x moments). The data of the slide readings were calculated, individually, through medians of the scores. To determine whether the frequencies of the median scores differed among experimental groups and the four moments, the Kruskal–Wallis non-parametric test was used. All analyzes were conducted using Bioestat 5.3 software, adopting a 5% significance level.

## Results

The results of CFI (g) and percentage WG in the groups supplemented with MOS were found to be lower than in the control group (p>0.05). The mean WC (mL) in the groups supplemented with MOS was higher than in the control group (p>0.05) (Table-2).

**Table-2:** CFI (g), percentage WG-%, and average WC (mL) of treatments/diets: Control (C), MOS1, MOS 2, and MOS 3, in rats in growth phase. Values presented as mean±standard deviation (n=8 group).

Parameters^†^	Treatments/diets^‡^			

	Control	MOS 1	MOS 2	MOS 3
CFI	1053.71 ±38.85	994.76 ±40.51	977.97± 58.51	976.92± 60.66
WG %	550.5± 12.1	543.6± 11.7	540.8± 12.3	536.9± 9.0
WC	26.43± 5.21	27.42± 4.98	27.87± 5.43	27.61± 4.75

† There was no statistically significant difference between groups by analysis of variance at 5% probability.

‡ Control and experimental diets/groups: Control (C): Basal diet (SD); MOS 1, SD diet supplemented with 1% of the MOS 1; MOS 2, SD diet supplemented with 4 g.Kg^-1^ of the MOS 2; and MOS 3, SD diet supplemented with 10 g.Kg^-1^ of the MOS 3. MOS=Mannan-oligosaccharides, CFI=Cumulative food intake, WG=Weight gain, WC=Water consumption

**Figure-2:**
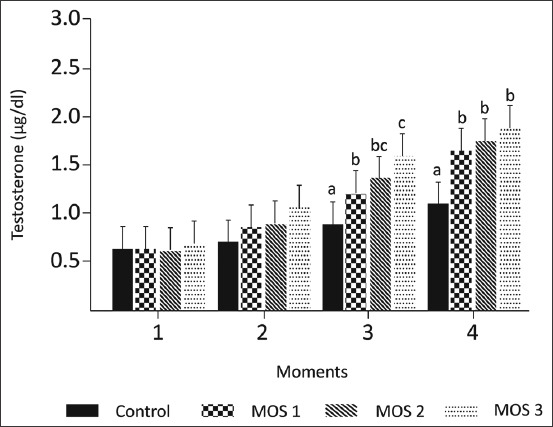
Histograms showing the plasma testosterone concentrations (μg/dL) at the four sample moments (1 [14 days]; 2 [28 days]; 3 [42 days]; and 4 [56 days]), of rats in the growth phase submitted to treatment/control diets and with three different mannan-oligosaccharides. One-way analysis of variance with Tukey’s test during each moment (p<0.05).

There were significant differences in the effect of the diet (p<0.05) on the weight of the adrenal glands only at moments 1 and 2. However, there were significant differences for diet effect and moment effect (p<0.05), no statically differences for moment interaction on the weight of the testicles were seeing (p>0.05) (Table-3). However, the groups supplemented with MOS presented higher concentrations of testosterone at moments 3 and 4 of the experimental period (p<0.05) (Figure-2).

**Table-3:** Effects of treatments/diets with MOS (prebiotics), at four sampling moments, on the weight of the adrenal glands and testicles of rats in the growth phase (means±SD).

Parameters	Diets/groups^††^	Moments^†^			

		1	2	3	4
Adrenal glands (g)	Control	0.0357±0.0039	0.0343±0.0067	0.0350±0.0051	0.0361±0.0023
	MOS1	0.0450±0.0127	0.0445±0.0065	0.0434±0.0094	0.0432±0.0087
	MOS2	0.0461±0.0079	0.0449±0.0054	0.0448±0.0081	0.0438±0.0091
	MOS3	0.0525±0.0127	0.0454±0.0059	0.0452±0.0056	0.0443±0.0057
Testicles (g)	Control	0.4752±0.0643	1.2584±0.1054	1.6894±0.1540	2.1884±0.1619
	MOS1	0.5563±0.0727	1.3272±0.1784	1.7663±0.1221	2.2572±0.1254
	MOS2	0.5627±0.0709	1.3392±0.1795	1.7820±0.1239	2.3692±0.1736
	MOS3	0.5695±0.0505	1.3910±0.1773	1.8463±0.1660	2.4090±0.1949

† Sample moments: 1 (14 days); 2 (28 days); 3 (42 days); and 4 (56 days) after the beginning of the experimental period. There was no significant difference between the groups, in each moment, using the Tukey test at 5% probability.

‡ Control and experimental diets/groups: Control (C): basal diet (SD); MOS 1, SD diet supplemented with 1% of the MOS 1; MOS 2, SD diet supplemented with 4 g.Kg^-1^ of the MOS 2; and MOS 3, SD diet supplemented with 10 g.Kg-1 of the MOS 3. MOS=Mannan-oligosaccharides

**Figure-3:**
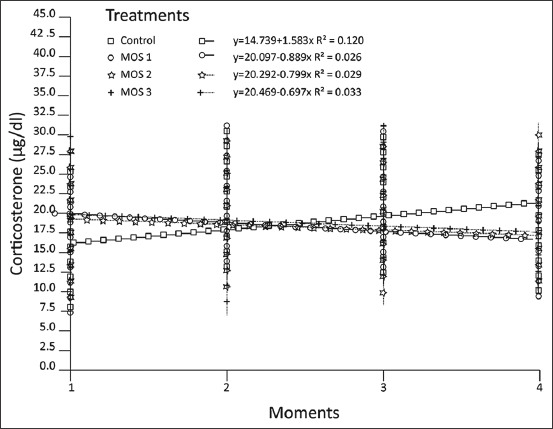
Evolution of plasma corticosterone concentrations (μg/dL) at the four sample moments (1 [14 days]; 2 [28 days]; 3 [42 days]; and 4 [56 days]), of rats in the growth phase submitted to treatment/control diets and with three different mannan-oligosaccharides (p<0.05).

The simple linear regression of the evolution of the corticosterone concentrations as a function of time showed a significant difference (p<0.05) between the angular coefficients of the analyzed lines (Beta), between the groups supplemented with MOS and control. The evolution of corticosterone concentrations in the treatment/control diet increased compared to those treated with different MOS (Figure-3).

The results of the frequencies of the spermatids and spermatozoa relative scores of the histological sections of the testicles are shown in Tables-4 and 5. In the parameter frequency of spermatids relative score, the statistical analysis revealed significant differences between the groups supplemented with MOS and the control group (p<0.01) at moments 1, 2, and 3 (p<0.05), while at the moment 4 no statistical differences were found between the groups supplemented with MOS and the control group (p>0.05). The results of the frequencies of spermatozoa relative score at moments 2, 3, and 4 of the supplemented groups differed significantly (p<0.05) from the control group.

**Table-4:** Frequency (%) of the relative scores for the number of spermatids, in histological sections (n=30) of the testicle of rats in the growth phase submitted to four different diets and at four sample moments.

Treatments/diets	Moments																p-value*

	1 (Scores^†^ at 14 days) (%)				2 (Score from day 15-28 days) (%)				3 (Score from day 29-42 days) (%)				4 (Score from day 43-56 days) (%)				
			
	0	1	2	3	0	1	2	3	0	1	2	3	0	1	2	3	
Control	30/30 (100)	0/30 (0)	0/30 (0)	0/30 (0)	15/30 (50)	15/30 (50)	0/30 (0)	0/30 (0)	0/30 (0)	0/30 (0)	12/30 (40)	18/30 (60)	0/30 (0)	0/30 (0)	0/30 (0)	30/30 (100)	<0.01
MOS 1	30/30 (100)	0/30 (0)	0/30 (0)	0/30 (0)	0/30 (0)	15/30 (50)	15/30 (50)	0/30 (0)	0/30 (0)	0/30 (0)	12/30 (40)	18/30 (60)	0/30 (0)	0/30 (0)	0/30 (0)	30/30 (100)	<0.01
MOS 2	6/30 (20)	24/30 (80)	30/30 (0)	30/30 (0)	0/30 (0)	0/30 (0)	21/30 (70)	3/30 (30)	0/30 (0)	0/30 (0)	0/30 (0)	30/30 (100)	0/30 (0)	0/30 (0)	0/30 (0)	30/30 (100)	<0.01
MOS 3	6/30 (20)	24/30 (80)	30/30 (0)	30/30 (0)	0/30 (0)	0/30 (0)	6/30 (20)	24/30 (80)	0/30 (0)	0/30 (0)	0/30 (0)	30/30 (100)	0/300)	0/30 (0)	0/30 (0)	30/30 (100)	<0.01
p-value**	<0.01				<0.01				<0.01				1.00				

* =Significant values in the Kruskal–Wallis test for comparisons between treatments.

** =Significance values in the Kruskal-Wallis test for comparisons between moments.

† The relative scores were classified from 0 to 3, from 30 microscopic fields, with 0=absence in 30 fields; 1=mild (1-10 out of 30 fields); 2=moderate (11-20 out of 30 fields); and 3=accentuated (>20 out of 30 fields) and were transformed into frequency (%). MOS=Mannan-oligosaccharides

**Table-5:** Frequency (%) of the relative scores for the number of spermatozoa, in histological sections (n=30) of the testicle of rats in the growth phase submitted to four different diets and at four sample moments.

Treatments/diets	Moments																p-value*

	1 (Scores† at 14 days)				2 (Score from day 15 to 28 days)				3 (Score from day 29 to 42 days)				4 (Score from day 43 to 56 days)				
			
	0	1	2	3	0	1	2	3	0	1	2	3	0	1	2	3	
Control	30/30 (100%)	0/30 (0%)	0/30 (0%)	0/30 (0%)	(27/30) (90%)	(3/30) (10%)	0/30 (0%)	0/30 (0%)	0/30 (0%)	18/30 (60%)	12/30 (40%)	0/30 (0%)	0/30 (0%)	0/30 (0%)	24/30 (80%)	6/30 (20%)	<0.01
MOS 1	30/30 (100%)	0/30 (0%)	0/30 (0%)	0/30 (0%)	6/30 (20%)	24/30 (80%)	0/30 (0%)	0/30 (0%)	0/30 (0%)	12/30 (40%)	12/30 (40%)	6/30 (20%)	0/30 (0%)	0/30 (0%)	9/30 (30%)	21/30 (70%)	<0.01
MOS 2	24/30 (80%)	6/30 (20%)	0/30 (0%)	0/30 (0%)	0/30 (0%)	21/30 (70%)	9/30 (30%)	0/30 (0%)	0/30 (0%)	9/30 (30%)	9/30 (30%)	12/30 (40%)	0/30 (0%)	0/30 (0%)	12/30 (40%)	18/30 (60%)	<0.01
MOS 3	24/30 (80%)	6/30 (20%)	0/30 (0%)	0/30 (0%)	0/30 (0%)	15/30 (50%)	15/30 (50%)	0/30 (0%)	0/30 (0%)	3/30 (10%)	9/30 (30%)	18/30 (60%)	0/30 (0%)	0/30 (0%)	0/30 (0%)	30/30 (100%)	<0.01
p**	0.58	<0.01	0.02	<0.01	

* =Significant values in the Kruskal–Wallis test for comparisons between treatments.

** =Significance values in the Kruskal-Wallis test for comparisons between moments.

† The relative scores were classified from 0 to 3, from 30 microscopic fields, with 0=absence in 30 fields; 1=mild (1-10 out of 30 fields); 2=moderate (11-20 out of 30 fields); and 3=accentuated (>20 out of 30 fields) and were transformed into frequency (%). MOS=Mannan-oligosaccharides

**Figure-4:**
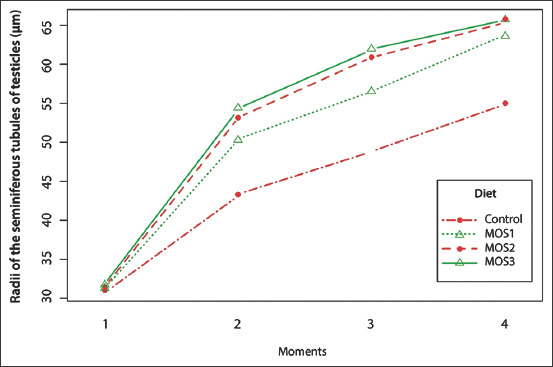
Interaction plot for effects of diets (control, mannan-oligosaccharides [MOS] 1, MOS2 and MOS3) and moments (1=14 days; 2=28 days; 3=42 days; and 4=56 days) on seminiferous tubules of testicles of rats in the growth phase after the beginning of the experimental period. Control and experimental diets/groups: Control with basal diet (SD); MOS 1, SD diet supplemented with 1% of the MOS 1; MOS 2, SD diet supplemented with 4 g.Kg^–1^ of the MOS 2; and MOS 3, SD diet supplemented with 10 g.Kg^–1^ of the MOS 3. Different letters indicate significant statistical differences.

The radius of the seminiferous tubules presented a significant statistical effect of the diet, moments, and diet + moment interaction (Table-6). When comparing the diets, it was found that the MOS showed significant effects on radii of the seminiferous tubules, with higher averages for MOS 3, followed by MOS 2 and MOS1. The interaction among diets and moments is shown in Figure-4.

**Table-6:** Measurements of the radii of the seminiferous tubules of testicles of rats in the growth phase supplemented with different MOS. Values expressed as mean±standard deviation.

Groups	Moments^†^/Seminiferous tubules (µm)			

	1	2	3	4
Control	30.62±0.99^A^	43.12±1.32^B^	48.73±1.01^C^	54.83±1.86^E^
MOS 1	30.90±0.76^A^	50.21±1.85^CD^	56.44±1.00^E^	63.65±2.76^FG^
MOS 2	31.07±0.71^A^	53.11±1.30^DE^	60.96±2.05^F^	65.57±3.32^G^
MOS 3	31.37±0.71^A^	54.29±1.23^E^	61.95±1.91^F^	65.72±3.15^G^

† Sample moments: 1 (14 days); 2 (28 days); 3 (42 days); and 4 (56 days) after the beginning of the experimental period. ^‡^Control and experimental diets/groups: Control (C): basal diet (SD); MOS 1, SD diet supplemented with 1% of the MOS 1; MOS 2, SD diet supplemented with 4 g.Kg^-1^ of the MOS 2; and MOS 3, SD diet supplemented with 10 g.Kg^-1^ of the MOS 3. Data were analyzed by two-way analysis of variance with a Tukey *post hoc* test at 5% probability. Different letters indicate significant statistical differences. MOS=Mannan-oligosaccharides

## Discussion

The supplementation with different MOS did not alter the feeding behavior, food intake, or WG of the rats. Studies in rodents have demonstrated that supplementation with fermentable dietary fiber in the diet can form a link between the result of microbial fermentation in the lower part of the intestine and the metabolic consequences, such as decreased food intake and WG, due to the beneficial modulation of secretion of intestinal peptide similar to glucagon1, glucose-dependent tropic insulin polypeptide, YY peptide, and/or ghrelin [19]. Other studies also did not observe a decrease in food intake or WG in rats with a weight considered healthy after prebiotic, supplementation in the diet [20,21], as well as in humans [22,23]. However, in obese or overweight rodents, prebiotics have been shown to assist in weight control by decreasing food intake [24].

WC did not alter with the inclusion of different MOS in the diet, as demonstrated in other studies with the use of the probiotics galursan HF 7K, B-GOS®, Larch gum (from *Larix occidentalis*), inulin, and oligofructose [23].

The weight of the adrenal glands had no effect on time, moments, or diet. However, the results revealed the effect of diet and time on testicle weight. Although only the weight of the testicles analyzed did change with the supplementation of MOS in the diet, the evaluations for the plasmatic levels of corticosterone and testosterone hormones were beneficially modified in plasma from the groups supplemented with the three different MOS. This may be the result of the indirect effect of these indigestible compounds, through the beneficial modulation of the amounts of beneficial bacteria classified as probiotics (mainly of the genera *Bifidobacterium* and *Lactobacillus*), in the complex community of microorganisms that resides in the intestinal tract of mammals [3,25]. These probiotic bacteria process insoluble dietary fibers, prebiotics, into SCFAs that can be directly or indirectly involved in communication along the microbiota-gut-brain axis due to their neuroactive properties and their effects on other intestinal-brain signaling pathways, including the immune and endocrine systems [25,26]. In addition, many studies demonstrated that testosterone in male rats was significantly predictive by testicular weight [27] and similar associations between testis size and plasma testosterone levels have been reported in other animal studies [27-29].

On the other hand, when there is an imbalance in the intestinal microbial community due to diet or stress, bacterial species such as Gram-negative bacteria and those with pathogenic capacity to cause endotoxemia represent a potentially significant threat to the health of the host [7]. At present, there are no experimental data that support a direct link between exposure to endotoxins in humans and impaired testosterone or spermatogenesis production. However, studies in women have confirmed an association between endotoxemia and a reduction in the ability of the ovaries to produce the sex steroid hormone progesterone [30,31]. In addition, there is evidence in animals suggesting that endotoxins have the ability to impair testicular function [32]. Therefore, the increase in plasma testosterone concentration may be due to the ability of prebiotics and probiotics to reduce circulating endotoxins and improve related sequelae, such as inflammation and or negative health markers [33].

A reduction in plasma corticosterone levels was also observed in the groups treated with MOS. This beneficial action of MOS prebiotics, classified as psychobiotics, is probably due to them serving as food for beneficial bacteria (probiotics) and influencing the relationship between bacteria and the brain. Psychobiotics applied to rodent models of infections provide early clinical information about human diseases and have revealed that abnormal intestinal microbiota can induce behaviors associated with the hypothalamic-pituitary-adrenal (HPA) axis and response to inflammatory processes that are generally characterized by aberrant concentrations of cytokines [34]. Glucocorticoids (cortisol in humans and corticosteroids in rodents) can suppress the hypothalamic-pituitary-gonadal axis, inhibiting the secretion of gonadotropin-releasing hormone from the hypothalamus and consequently suppressing the release of gonadotropin from the pituitary and the synthesis of sex hormones in gonads, culminating in the reduction or elimination of reproduction and sexual behavior [35,36], mainly in stress situations [37].

The results revealed anticipation of an increase in the frequency of spermatids in groups supplemented with prebiotics in the diet. This postnatal sexual development of male rats occurs in four phases: A neonatal period from birth to 7 days of age; a postnatal childhood period from 8 to 21 days; an adolescent period that extends to approximately 35 days of age; and a peripubertal period up to 55-60 days of age which ends when mature sperm are seen in the vas deferens [38]. This anticipation in the number of spermatids may be due to the indirect action of the increase in the hormone testosterone, allowing the germ cells to complete meiosis [39].

The results also revealed an increase in the number of spermatic cells, spermatozoa, in the groups supplemented with MOS. This finding is consistent with previous studies in which glucocorticoids affect spermatogenesis and can cause meiotic arrest in germ cells, since glucocorticoids have receptors located in Leydig cells and in spermatocytes in meiosis prophase I [40,41]. The regulation of spermatogenesis depends on FSH and testosterone. While FSH plays a major role in the development of the immature testicle, causing the proliferation of Sertoli cells and the progression of spermatogonia A to spermatogonia B, testosterone alone can maintain complete spermatogenesis [42]. FSH and testosterone have receptors on Sertoli cells and modulate spermatogenesis by regulating the function of Sertoli cells [43].

Together these data indicate an acceleration of puberty in male rats treated with MOS prebiotics due to the change in the steroidogenic axes and the increased spermatogenesis at 51 and 65 days of age. This may also be due to the decrease in corticosterone levels, which have an inhibitory effect on reproduction by intervening in the HPA axis [35], since corticosterone acts on the intracellular receptors that interact with transcription factors and is capable of regulating gene expression of enzymes and causing a variety of responses [39].

We also observed greater development of seminiferous tubules in the groups treated with MOS, which reinforces the hypothesis that treatment with the prebiotic accelerated the sexual maturity of the rats. A study demonstrated that glucocorticoids negatively influence puberty of male rats, since the application of dexamethasone led to a disorder in the morphology of seminiferous tubules, presenting an irregular, and disorganized basal membrane in some areas, with stratified germinal epithelium cells showing vacuoles in their cytoplasm. The period of puberty is critical and corticosterone has a great influence in this phase on the secretion of testosterone and in the proliferation of Sertoli and germ cells [42].

These results have important clinical relevance since the MOS prebiotic in pubertal rats led to a change in steroidogenesis, representing a decrease in cortisol and an increase in plasma testosterone, improving the reproductive profile, verified by the higher proportion of spermatids and spermatozoa in treated animals and greater development of seminiferous tubules. In view of the results observed, we suggest that further studies should be carried out in adult and older rats, seeking improvement in the endocrine and reproductive profile in humans to bring benefits to the health of individuals.

Other studies report that supplementation with prebiotics demonstrated direct benefits, for example, carbohydrate structures similar to host glycans can block adhesion to host cells [44] or indirect benefits by encouraging the proliferation of intestinal microbiota bacteria whose metabolites directly affect the intestinal environment or host gene expression, for example, the effect of SCFAs on intestinal pH and on the modulation of immune functions and regulation of axis development (HPA) [45,46]. The role of the intestinal microbiota was evidenced in germ-free mice, where mild restrictive stress induced an exaggerated release of corticosterone and adrenocorticotrophic hormone compared to specific pathogen-free controls. This has since been replicated independently, again after a mild stressor, both in mice [47] and in rats [48].

Although the rats used in the current experiment were not purposely exposed to stressful procedures, all animals were regularly subjected to routine maintenance or monitoring procedures, such as personnel entering the animal room, moving about, and cleaning cages, feeding and fasting cycles, body weight collection, and physical examination, and although these procedures can be considered incidental so that their effects on the welfare of laboratory animals can be neglected by the ethical review committees, these routine procedures can cause significant stress to animals, although apparently benign [49]. Studies report that handling murines leads to a significant increase in blood corticosterone concentration [50,51], as well as changing the cage [50] and the environment [52]. Such hypothesis seems to be strengthening by the work of Mucignat-Caretta *et al*. that demonstrated how young mice are more responsive to mild stressors, such as environmental ones, with higher corticosterone plasmatic peaks [53]. Corticosterone, a result of the rapid activation of the HPA axis, is considered a gold standard biomarker of acute stress [54,55].

Finally, environmental factors such as diet, drugs (e.g., antibiotics), pathogens, and toxic and environmental pollutants can directly and indirectly stress the intestinal microbiota, through alterations in inflammation, oxidative stress, immune function, and the GI environment. The composition of the diet, in particular, is an important factor that influences the intestinal microbiota, due to the intake of nutrients that directly affect the types and nutrients available to the intestinal microbes and the countless effects of different nutrients on the physiology of the host [56,57]. All animals were exposed to the same possible stressors, which should result in the same blood concentrations of corticosterone; however, the groups supplemented with the three different MOS demonstrated a significant reduction in corticosterone, indicating a beneficial effect in response to these possible stressor agents.

In addition, the radii of the seminiferous tubules showed greater development in the groups treated with MOS. These results confirm the previous data and reinforce the hypothesis that treatment with the prebiotic accelerated the sexual maturity of the rats. A study demonstrated that hormones of the glucocorticoid class, such as corticosterone, negatively influence the puberty of male rats and lead to a disorder in the morphology of the seminiferous tubules, presenting an irregular and disorganized basal membrane in some areas, with stratified germinal epithelium cells presenting vacuoles in their cytoplasm. The period of puberty is critical and corticosterone has great influence in this phase in the secretion of testosterone and in the proliferation of Sertoli and germ cells [42]. Therefore, the decrease in blood corticosterone concentration observed in the current study in groups supplemented with the three different MOS may be responsible for the increase in testosterone and, indirectly, in the maturation of seminiferous tubules.

These results have important clinical relevance since the prebiotic MOS led to a change in steroidogenesis in pubertal rats, representing a decrease in cortisol and an increase in plasma testosterone, improving the reproductive profile, verified by the higher proportion of spermatids and spermatozoa in treated animals and greater development of seminiferous tubules. In view of the observed results, we suggest that other studies should be carried out in adult and older rats, seeking improvement in the endocrine and reproductive profile in humans to bring benefits to the health of individuals.

## Conclusion

It was concluded that the three different MOS prebiotics tested in this experiment anticipated sexual maturity in growing Wistar rats through higher plasma concentrations of testosterone and reduced cortisol, as well as increased spermatid production and, consequently, higher spermatozoa production.

## Authors’ Contributions

HB: Made substantial contributions to the conception or design of the work, preparation of diets, the acquisition, analysis, and interpretation of data; drafted the work, and approved the submitted version. LER: Made substantial contributions to the analysis and interpretation of data; drafted the work and approved the submitted version. MMK: Made substantial contributions to the conception or design of the work and animal management. RK: Made substantial contributions to the design, analysis and interpretation of data, and approved the submitted version. HRSC: Made substantial contributions to the acquisition, analysis and interpretation of corticosterone and testosterone, and approved the submitted version. MNR: Made substantial contributions to the acquisition, analysis and interpretation of data measurements, and approved the submitted version. OCS: Made substantial contributions to the histological analysis and interpretation of data and approved the submitted version. RG: Made substantial contributions to the analysis and interpretation of data and approved the submitted version. ICG: Made substantial contributions to the conception or design of the work and interpretation of data; drafted the work, and approved the submitted version. All authors have read and approved the final manuscript.
